# Development of a 3-D Physical Dynamics Monitoring System Using OCM with DVC for Quantification of Sprouting Endothelial Cells Interacting with a Collagen Matrix

**DOI:** 10.3390/ma13122693

**Published:** 2020-06-12

**Authors:** Yong Guk Kang, Hwanseok Jang, Yongdoo Park, Beop-Min Kim

**Affiliations:** 1 Department of Bio-Convergence Engineering, College of Health Science, Korea University, Seoul 02841, Korea; kygwow@korea.ac.kr; 2 Department of Biomedical Sciences, College of Medicine, Korea University, Seoul 02841, Korea; kevin14@korea.ac.kr

**Keywords:** OCT, OCM, SHG, DVC, cell–ECM physical interaction, vasculogenesis

## Abstract

The extracellular matrix (ECM) plays a key role during cell migration, proliferation, and differentiation by providing adhesion sites and serving as a physical scaffold. Elucidating the interaction between the cell and ECM can reveal the underlying mechanisms of cellular behavior that are currently unclear. Analysis of the deformation of the ECM due to cell–matrix interactions requires microscopic, three-dimensional (3-D) imaging methods, such as confocal microscopy and second-harmonic generation microscopy, which are currently limited by phototoxicity and bleaching as a result of the point-scanning approach. In this study, we suggest the use of optical coherence microscopy (OCM) as a live-cell, volumetric, fast imaging tool for analyzing the deformation of fibrous ECM. We optimized such OCM parameters as the sampling rate to obtain images of the best quality that meet the requirements for robust digital volume correlation (DVC) analysis. Visualization and analysis of the mechanical interaction between collagen ECM and human umbilical vein endothelial cells (HUVECs) show that cellular adhesion during protrusion can be analyzed and quantified. The advantages of OCM, such as fine isotropic spatial resolution, fast time resolution, and low phototoxicity, make it the ideal optic tool for 3-D traction force microscopy.

## 1. Introduction

The extracellular matrix (ECM) is a three-dimensional (3-D) natural polymer surrounding cells and tissues that not only maintains the architecture of our bodies and organs but also provides biochemical and biophysical signaling cues to adjacent cells. In particular, the biochemical composition and physical properties of the ECM play a pivotal role in diverse biological processes, such as development [1], wound repair [2], and even in the progression of diseases, including cancer [3,4,5,6,7,8]. In terms of its biochemical composition, the ECM provides adhesive substrates for cells, instructs cells to differentiate into appropriate cell types, and degrades itself in the formation of organs by delineating their spatial compartments during development and regeneration [1]. In terms of its physical properties, the generation of stiffer tissues during wound healing and solid tumor formation provides physical cues to cells, inducing downstream signals through a process called mechanotransduction. Therefore, monitoring and analyzing the localization and deformation of the ECM, both spatially and temporally, are critical to understanding the interaction of the ECM with surrounding cells.

Technologies for visualizing the distribution of specific proteins in the ECM have been developed and adopted as conventional analysis techniques, and include immunohistochemistry, immunofluorescent images, as well as scanning electron microscopy. However, since these methods require sample fixation and post-treatment processes for observation, is not possible to visualize the distribution and deformation of the ECM over time, and novel technologies using optics are required. Monitoring of the ECM over time has been achieved using optical techniques, such as in the imaging of collagen type I with second-harmonic generation and fluorescently labeled fibronectins. Moreover, observing the 3-D structure of the ECM requires the use of optical microscopy methods with depth sectioning ability, such as confocal laser scanning microscopy [9,10], confocal reflectance microscopy [11,12], or multiphoton microscopy. However, because these approaches use point-scanning methods, they are generally too slow for imaging rapid dynamics. To overcome this limitation, video-rate imaging with depth sectioning ability, such as light-sheet microscopy [13,14] or optical coherence tomography [15,16], is a promising technology.

Optical coherence tomography (OCT) is a low-coherence interferometry-based imaging technique that can acquire tomographic images of biological tissues without pretreatment by using near-infrared (NIR) light. A typical advantage of the OCT system is that it can generate images with a high adjustable range of temporal and spatial resolution [16,17,18,19]. Therefore, optical coherence microscopy (OCM)—in which the OCT technique is applied to a microscope system—can monitor micro-sized structures of cells and surrounding fibrous scaffolds in 3-D without the need for labeling [20,21,22,23]. One of the key determinants of the imaging quality in all coherent imaging techniques, including OCM, is speckles, which are derived from the interferometric sum of coherent illumination over the rough surface or scattering volume of the imaging media [24,25,26,27]. Speckles not only reflect the characteristics of the media being imaged but also have the characteristics of noise. Nevertheless, if the imaging conditions are optimized toward reducing aliasing and decorrelation of the speckles to obtain the correlation of volumetric images over time, image-based quantitative analysis, such as for tracking the deformation of the medium, is possible. Digital volume correlation (DVC) is the most well-known image-based computational process for quantifying 3-D full-field deformation in experimental mechanics [28]. The algorithm calculates the 3-D displacement between consecutive volume images by finding the value of shifted voxels that maximize cross-correlation between subvolumes of the images. In particular, even though in tissue, physiologically relevant ECM components such as collagen, fibrin, and laminin have nonlinear stress–strain relations due to their fiber structures [29,30], advanced nonlinear modeling allows for the analysis of their mechanical deformation [31].

Vascular endothelial cells (ECs) are formed along the inner wall of the blood vessel and act as a barrier to transport or selectively transfer substances in the blood [32]. In vitro studies in applied biology and experimental biology have widely used ECs as an experimental model to verify vasculogenesis and angiogenesis [33,34,35]. ECs are characterized by dynamic physical interaction with ECM structures in the 3-D environment during active branching and networking [36,37]. Although the aforementioned system is required to study the dynamic physical interaction between ECs and the ECM, it has been difficult to observe living cells in a 3-D matrix to date due to problems resulting from long-term imaging and phototoxicity. Therefore, this study aims to understand cell–ECM physical interactions by developing and optimizing an OCM system that can visualize the structure of collagen type I and applying a DVC algorithm that can quantify the physical deformation of the ECM by cells. Furthermore, this system is applied to measure the physical interactions between a sprouting EC and the ECM, which are important factors for understanding angiogenesis and development, and to visualize the physical communications between distant cells through the ECM medium.

## 2. Materials and Methods

### 2.1. Imaging System

#### 2.1.1. Gabor-Domain Optical Coherence Microscope

The basic setup of the optical imaging system was based on a commercial upright microscope (BX-51, Olympus, Tokyo, Japan). To implement the high-resolution spectral domain (SD) GD-OCM system, a broadband superluminescent diode (SLD) (T-850-HP-I, Superlum, USA) and spectrometer (Cobra-S, Wasatch, UT, USA) were used with a 20× water immersion objective lens (full NA 0.5, Olympus, Tokyo, Japan). An objective lens positioner (MIPOS500, Piezojena, Jena, Germany) was used for scanning focus in the optical axis direction. The step size of the objective lens position was set to half of the Rayleigh length of Gaussian focus. Analog voltage-controlled galvanometer mirror pairs (GVS101, Thorlabs, Newton, NJ, USA) were positioned with 4-f configuration to control the position of scanning focus in an optical transverse direction. A three-axis linear actuator-based (LTA-HS, LTA-HL, ESP301, Newport, Irvine, CA, USA) custom-built stage (ST1, Incheon, Korea) with a stage-top live-cell imaging chamber (Chamlide, Live cell instruments, Seoul, Korea) was used to maintain stable environmental conditions during imaging. A period of 30 min of idle time was implemented for every experiment after placing the chip on the stage to eliminate drifts due to thermal expansion and actuator backlashes.

#### 2.1.2. Second-Harmonic Generation Microscope

Second-harmonic generation microscopy (SHGM) was implemented by sharing the scanning beam path of GD-OCM. An ultrafast laser (Chameleon Ultra-II, Coherent, Santa Clara, CA, USA) and alkali photomultiplier tube (PMT) (H10862-210P, Hamamatsu Photonics, Hamamatsu, Japan) were used as the source and detector, respectively. A custom-built condenser lens with a dichroic filter cube was attached under the specimen stage to detect forward-scattered SHG [38]. A 2.5× beam expander was positioned in front of the ultrafast laser to match the required NA for SHGM. A Glan laser polarizer with waveplates was used to linearly control the incident power. 

### 2.2. Image Acquisition Process

#### 2.2.1. SHGM Image Acquisition

SHG images were acquired through a custom-written program based on C++/CLI in the Windows 10 (Microsoft, Albuquerque, NM, USA). Custom-built MATLAB (R2019b, Mathworks, Natick, MA, USA) scripts were used to merge, correct drift, and analyze the images as necessary.

#### 2.2.2. OCM Image Acquisition and Postprocessing

OCM images were acquired through a custom-written program based on C++ with Compute unified device architecture (CUDA), and the linear stage and objective lens positioner were controlled via serial communication. Reconstruction of OCT images followed a basic SD -OCT image processing protocol [39,40]. Zero-padding in k-space spectrum data were applied to increase the sampling rate of the axial dimension. The RANSAC [41] plane-fitting algorithm and Fourier shift theorem were used to fix the skewed optical pathlength over the image plane. Gabor-domain fusion was conducted at 10-micron intervals to achieve depth-invariant image quality in the axial direction. The fusion was carried out with trapezoidal windows [42,43]. All reconstruction and postprocessing was done using MATLAB.

### 2.3. Sample Preparation

#### 2.3.1. Microfluidic Chip Fabrication

Microfluidic chips were used to control the consistency of the collagen gel quality and quantity, including volume and density, using a design described in previous studies [44,45]. The microfluidic chip was fabricated with PDMS (Sylgard 184, Dow Corning, Midland, MI, USA) through a soft lithography process. The trimmed PDMS chip (25 mm × 25 mm square) was bonded with a cover glass by surface treatment of the lithographed face of the PDMS pieces and one side of the cover glass using an oxygen plasma treatment system to generate microchannels in the chip.

#### 2.3.2. Collagen Type I Gel Preparation

All collagen type I (Corning, New York, NY, USA) gel used in this study had a concentration of 2.5 mg/mL. The collagen solution was mixed with 10× phosphate-buffered saline (PBS) and was one-third distilled water with a neutral of pH 7.4 as adjusted using NaOH. The pre-cured collagen gel mixture was introduced into the channel of the prepared microfluidic chip and polymerized in a humidified 37 °C incubator. After 40 min of the gelation process, phosphate-buffered saline (PBS) or endothelial cell growth medium (EGM) was added to adjacent channels.

#### 2.3.3. Generating Strain with Nd Magnet and Steel Rod

For artificial strain measurement experiments, an Nd permanent magnet (ϕ: 2 mm, L: 3 mm) was located in the middle of the channel before collagen gel polymerization. A sliding mechanism with 3-axis optomechanical stages (Thorlabs, Newton, NJ, USA) was used to position a steel rod adjacent to the Nd magnet inside the channels. The magnetic attraction force between the Nd magnet and steel rod enabled the production of artificial strain over collagen gels without any physical touching. GD-OCM images were acquired before and after the magnetic attraction force was applied.

#### 2.3.4. HUVEC Preparation and Imaging

Human umbilical vein endothelial cells (HUVECs obtained from pooled donors, Lonza, Walkersville, ML, USA) were cultured and maintained in T75 cell culture flasks (SPL, Pocheon, Korea) containing EGM-2 media (Lonza, Walkersville, ML, USA) in a 37 °C and 5% CO_2_ environment. HUVECs were mixed with collagen gels before being injected into the microfluidic chip. Chips were flipped during the gelation process to set the HUVECs far from the walls of the channels. Vein endothelial growth factor (VEGF) was added to cell media to exert protrusion during live-cell imaging. Live-cell time-lapse GD-OCM imaging of HUVECs and their surroundings was performed with a time interval of 10 min. The data were saved as binary files and reconstructed after all imaging was completed to avoid storage overhead.

### 2.4. DVC Analysis

#### 2.4.1. Error Floor Evaluation

DVC analysis was conducted using the fast iterative DVC [46] method. The subset size was fixed to 128 pixels. Two consecutively scanned GD-OCM intensity volumes (volume 1 and volume 2) with different transverse sampling pixels (400, 600, and 800 pixels) were analyzed to compare the error floor. These cases correspond to sampling rates of 0.8, 0.53, and 0.4 μm/px due to the fixed transverse field of view (320 μm × 320 μm). Sampling rates over 0.8 μm/px or under 0.4 μm/px were not considered because the former does not meet the Nyquist rate, and the latter is computationally inefficient for DVC analysis (e.g., 0.2 μm/px sampling of an image volume with 16 Gabor stacks: 1600 × 1600 × 2048 × 16 pixels for each acquisition).

Virtually translated volumes were generated for each sampling rate. All samples were shifted with the same physical length but different voxel sizes. Four, six, and eight voxels were shifted for each sampling rate corresponding to a physical length of 3.20 μm in the transverse axis and 4.43 μm in the axial axis.

#### 2.4.2. Drift Correction and Calculation of the Incremental Displacement

Unwanted global drift in the measured displacement map is unavoidable. This drift comes from various sources, such as thermal expansion, gravitational force of the collagen gel, or the backlash of mechanical stages. To compensate for this, the mean value of each incremental displacement for each Gabor stack was regarded as the global drift for each depth [47]. Subtracting this global drift for every timestep and shift of GD-OCM image produces a drift-compensated image. After compensating for global displacements, cumulative displacement maps were calculated from incremental displacement data using this relation:

(1) $$cuU_{1}\left(x,y,z\right)=inU_{1}\left(x,y,z\right)$$

(2) $$cuU_{n}\left(x,y,z\right)=\mathrm{cu}U_{n-1}\left(x,y,z\right)+inU_{n-1}\left(cuU_{n-1}\left(x,y,z\right)\right)$$

where $inU_{n}$ and $cuU_{n}$ are the incremental and cumulative displacement maps at time n. For computation, the interpolation of the deformed grid mesh was used [48].

#### 2.4.3. Strain Calculation

From the definition of strain, the strain tensor is related to the deformation vector using:

(3) $$\varepsilon_{\mathrm{ij}}=\frac{1}{2}\left(\frac{\partial u_{i}}{\partial x_{j}}+\frac{\partial u_{j}}{\partial x_{i}}\right)$$

where $u_{i}$ denotes the deformation vector of the i-th axis component. This means that the gradient of the displacement map can produce strain maps. However, this direct differentiation is susceptible to random noise. The effect of random noises was reduced by mean filtering of the displacement matrix. Additionally, instead of direct differentiation, central differential methods with 3 × 3 × 3 voxel kernels were used in our study [28].

## 3. Results

### 3.1. GD-OCM System Configuration and Performance Verification

**Figure 1:**
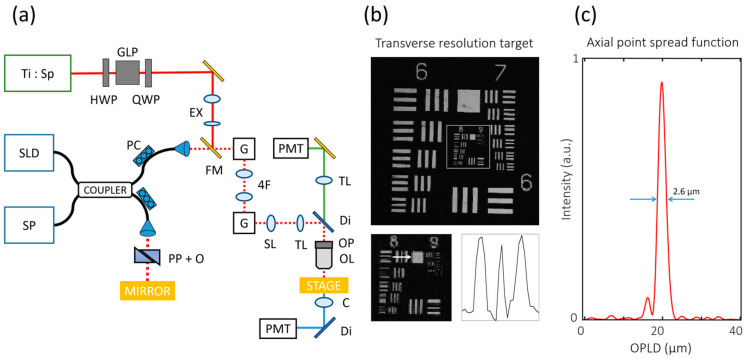
Graphical description of spectral domain Gabor-domain optical coherence microscopy (GD-OCM) system performance. (**a**) Schematics of GD-OCM with the second-harmonic generation microscopy (SHGM) system. (**b**) Transverse resolution measurement from the United states air force (USAF) 1951 resolution target. Element 1 in group 8 is the finest feature that can be resolved in both the X- and Y-axes. (**c**) The axial point spread function is 2.6 µm in water (zero-padded 8 times). (Ti:SP: titanium sapphire laser; SLD: superluminescent diode; SP: spectrometer; GLP: Glan laser polarizer; PC: polarization controller; EX: beam expander; FM: flip mirror; G: galvanometer mirror; SL: scanning lens; TL: tube lens; PP + O: prism pair with other dispersive optics; Di: dichroic mirror; OP: objective lens positioner; OL: objective lens; C: condenser lens; PMT: photomultiplier tube; OPLD: optical pathlength difference.).

The GD-OCM system was integrated into the SHGM used in our previous study [44] to visualize the ECM using two independent optical modalities (Figure 1a). Flip mirrors were used to switch between the two optical modalities. After that, the spatial resolution of GD-OCM was verified. Transverse resolutions were measured with the United states air force (USAF) 1951 resolution target (Edmund Optics, Barrington, NJ, USA). Element 1 in group 8 is the finest feature that can be resolved; this corresponds to 1.96 µm (Figure 1b). The axial point spread function (PSF) in water medium was measured from the image of the metallic first-surface mirror (Figure 1c). The measured full-width at half maximum (FWHM) of the PSF was 2.6 µm. As a result, we verified that images acquired using this OCM system had sufficient resolution for observing cells and the surrounding ECM.

**Figure 2:**
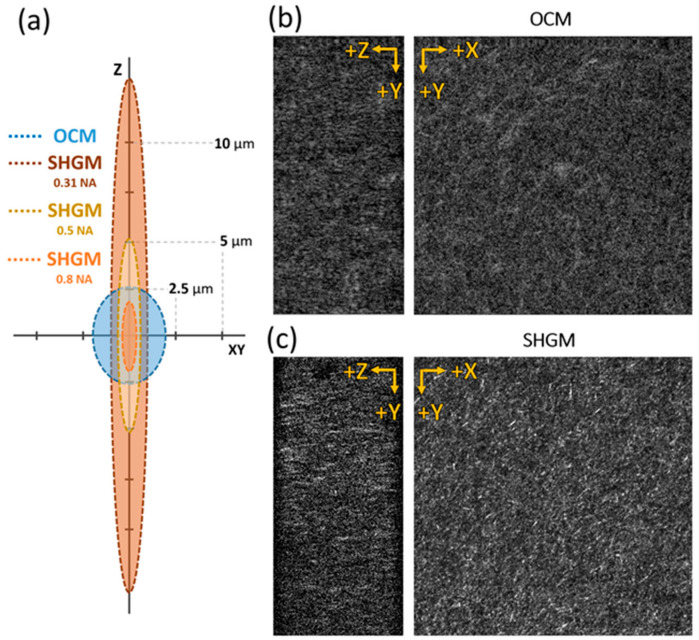
Cross-sectional and *en face* image of collagen fiber scaffolds (2.5 mg/mL). (**a**) Schematics of imaging point spread function (PSF) size in the axial (Z) and transverse (XY) axes for GD-OCM, and SHGM with different numerical aperture (NA) (0.31, 0.5, and 0.8 NA). (**b**) OCM images of collagen sample; (**b**) SHGM images of collagen sample. The cross-sectional ZY plane image in (**c**) shows a more anisotropic, elongated image than (**b**). The field of view is 320 × 320 × 160 µm^3^. Full-width at half maximum (FWHM) is used as the size of PSF.

Next, to compare the image of the collagen structure using GD-OCM and SHGM, 2.5 mg/mL collagen gel injected in a microfluid chip was imaged with the same field of view (320 × 320 × 160 µm^3^) (Figure 2). We used the same objective lens for both imaging modalities (20 × 0.5 NA water immersion, Olympus, Tokyo, Japan). The GD-OCM system in this study had a more isotropic optical resolution (FWHM of PSF) than SHGM (Figure 2a). The theoretical transverse and axial resolution of SHGM are estimated to be 0.98 and 13.85 µm, respectively [49] (the effective NA_SHG_ in this system is 0.31). The ratio of transversal resolution to axial resolution was 0.07 for GD-OCM and 0.75 for SHGM. GD-OCM showed 10 times better isotropic imaging capacity than SHGM. In addition, the resolutions of SHGM with 0.5 NA (maximum achievable NA of the objective lens) and 0.8 NA (40× objective lens used in the previous study) were also estimated, however SHGM with the higher NA still showed anisotropic imaging resolution between the transverse and axial axes. As a result, the isotropic resolution using the GD-OCM system works in favor of the operation of the DVC algorithm.

The total acquisition time of GD-OCM, including multiple Gabor stack acquisition, was approximately 5 min or less in this study. On the other hand, the acquisition time of SHGM was 10 min for the same volume, and the transversal sampling rate was rather low as the 512 × 512 pixels. The low acquisition time of GD-OCM means higher temporal resolution (2-times SHGM in this study) with a low probability of phototoxicity. Using our configuration, both GD-OCM and SHGM can image the collagen ECM while maintaining live-cell conditions; however, SHG is better at achieving a clear image of collagen structures in 2-D, while GD-OCM is better for isotropic, fast imaging over a long period.

### 3.2. Optimization of the Efficient Sampling Rate for Detecting Collagen Gel Structural Deformation

Various sampling rates were tested to find the optimum sampling rate that meets both the quality and efficiency needed for the DVC analysis. Transversal sampling rates were determined by adjusting the voltage steps of the galvanometric scanning mirror, while axial sampling rates were determined by adjusting the zero-padding rate before FFT operation during the GD-OCT image reconstruction process.

**Figure 3:**
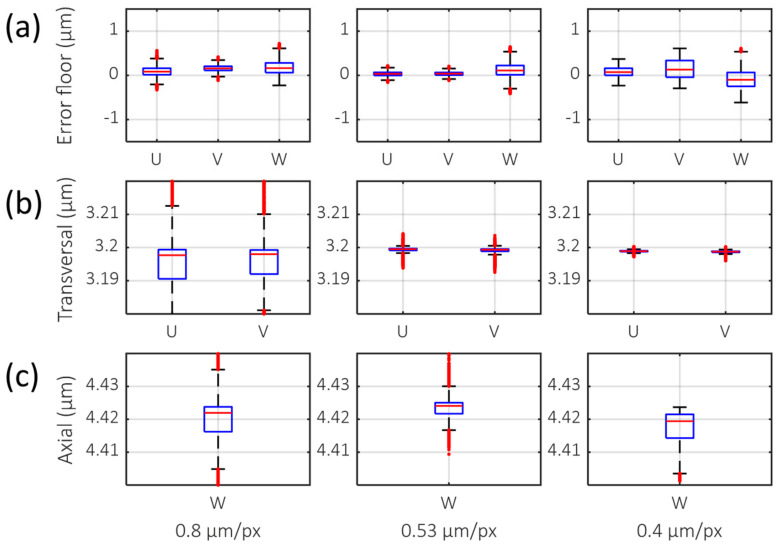
Boxplot of DVC analysis with various transversal sampling rates. U, V, and W indicate deformation in X-, Y-, and Z-directions, respectively. (**a**) Error floor and (**b**) virtual translation in the transverse axis, 3.20 µm in U and V. (**c**) Virtual translation in the axial axis, 4.42 µm in W. The red horizontal line is the median value. The top and bottom of blue box indicates the 25th and 75th percentiles. Red crossmark outside of the box denotes outlier.

An error floor (standard deviation and interquartile range of measured deformation value) was measured (Figure 3a) for three different transversal sampling rates to measure the sensitivity of DVC. The error floor measured (Table 1, standard deviation (STD)) indicates that 0.53 µm/px had the lowest error floor among test cases (0.083 µm), especially in the transverse direction (0.047 µm, 0.040 µm for U, V axis). The interquartile range (Table 1, Interquartile range (IQR)) also showed the same results. The accuracy of the displacement measurement was also measured (Figure 3b) with different transversal sampling rates. The displacement of the collagen gel was induced by the virtual shift of the voxel image. The mean value of the measured displacement for all sampling rates was 3.19 µm for transversal and 4.42 µm for axial (Table 1). The median (Indicated as red bar in Figure 3) of deformation also showed near identical results of mean. This matched well with the ground truth of the shifted values (3.20 µm, 4.42 µm). The accuracy of displacements with physically translated samples was also tested (Figure S1). A sampling rate of 0.53 µm/px showed minimal errors.

Various axial sampling rates were considered. As a result, a total length of 2048 pixels (with zero-padding of 1024 pixels) for a single A-line (1-D spectrum) was selected. This amount of zero-padding allowed a 0.73 µm/px sampling rate. This sampling rate is ideal for two reasons. First, its value is similar to the transverse sampling rate, so it was advantageous for isotropic imaging PSF. Second, a 2048-pixel length satisfies the power of two (2^n^) that can excel in the FFT algorithm, which is critical for efficiency of GD-OCT image reconstruction. As a result, we chose 0.53 µm/px for transversal and 0.73 µm/px for axial as the default sampling rate in this study because we believe that this provides the optimal balance between data size and accuracy.

### 3.3. Quantification and Visualization of Collagen Gel Deformation and Its Strain

**Figure 4:**
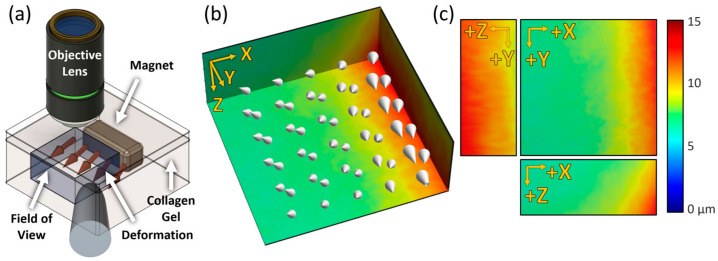
Mechanical stimulus using a magnet on the collagen gel and its visualization. (**a**) Schematics of applying an arbitrary force on the collagen gel. The blue cube indicates the imaging field of view. (**b**) The absolute magnitude of displacements depicted with a color-coded map and cone arrows over the field of view. (**c**) The magnitude of deformation for each axis (U corresponds to the *X*-axis, V corresponds to the *Y*-axis, and W corresponds to the *Z*-axis. Field of view is 224 × 224 × 130 µm^3^).

In order to deform the collagen gel structure without causing damage, a neodymium magnet was embedded in the matrix to generate external forces on the structure (Figure 4a). The position of the magnet was parallel to the YZ plane at the positive end of the *X*-axis in the field of view. The magnet was dragged by setting the tip of a steel rod at an arbitrary location, and the GD-OCM images were taken before and after the stimulus. Through DVC analysis, the magnitude and direction of displacement of the collagen structure were visualized with a 3-D color-coded map and cone arrows, respectively (Figure 4b). According to the direction and relative size of the cone arrows, the external force dragged the collagen gel structure in the negative Z-direction while pushing it in the negative X- and the positive Y-direction. As shown with the maximum intensity projection of each axis (Figure 4c), the largest displacement was around 15 µm on the side that contacted the magnet, and the farther away from the magnet, the smaller the displacement (below 6 µm). This means that our GD-OCM system and DVC analysis can quantitatively analyze the local deformation of the collagen matrix due to physical force and identify the direction and magnitude of an arbitrary external stimulus.

**Figure 5:**
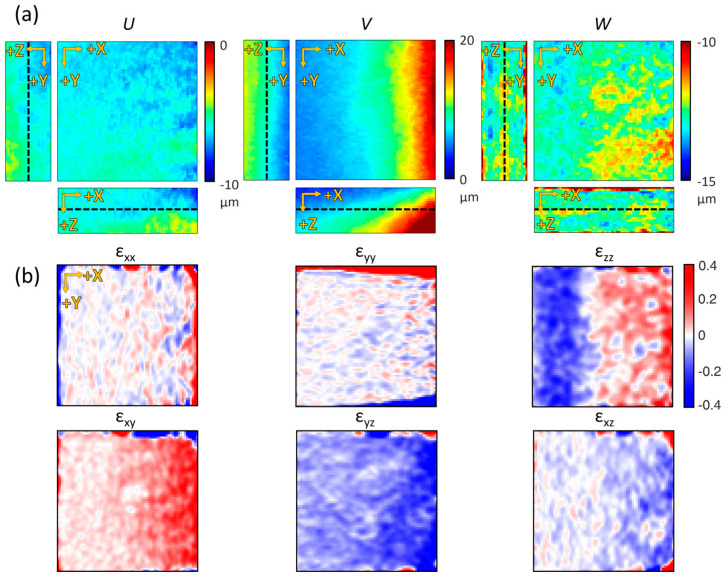
Displacement maps in each axis and their corresponding strain maps. (**a**) U, V, and W displacement map sliced along its center of volume. (**b**) Corresponding local strain maps. The selected Z-depth is the median of the axial field of view (indicated as black dashed lines in (**a**)) (all fields of view are 224 × 224 × 130 μm^3^).

To further understand the matrix deformation, the displacement and strain tensor were mapped by dividing the measured deformation result for each axis component (Figure 5). Since the degrees of movement in each direction were different, the displacements of each direction component—U, V, and W—were plotted in various ranges on the length scale (Figure 5a). The dashed lines in the panels with orthogonal view indicate the selected slices in the *Z*-axis plane. The displacements were measured from 0 to 10 μm in the negative direction along the *X*-axis, 0 to 20 μm in the positive direction along the *Y*-axis, and between 10 and 15 μm in the negative direction along the *Z*-axis. Normal strain ($\varepsilon_{xx}$, $\varepsilon_{yy}$, and $\varepsilon_{zz}$), calculated from the same plane as the displacement results (U, V, and W), showed extensible deformation near the magnet and contractile deformation away from the magnet (upper panels in Figure 5b). Shear strain ($\varepsilon_{xy}$, $\varepsilon_{yz}$, and $\varepsilon_{xz}$) showed that high distortion occurred near the magnet (lower panels in Figure 5b). As a result, we confirmed that the OCM imaging and DVC analysis were able to quantify and visualize the deformation of the collagen gel structure at microscopic resolution for every axis.

### 3.4. Observation of the ECM-Mediated Physical Dynamics of Sprouting Vascular Endothelial Cell and Distant Cells

**Figure 6:**
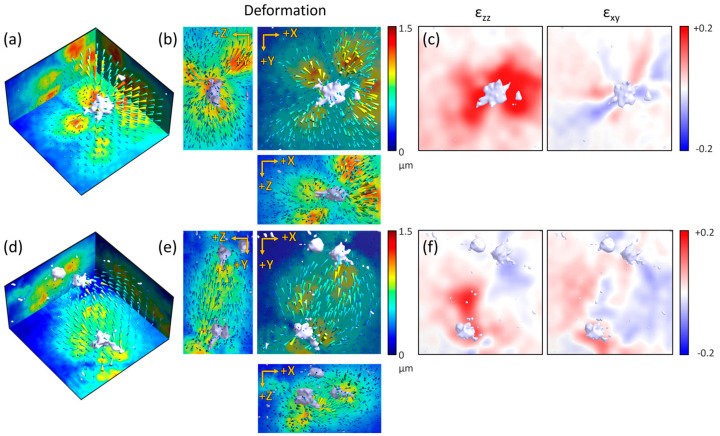
Visualization and analysis of the cell–ECM physical interactions of a single sprouting human umbilical vein endothelial cell (HUVEC) and two distant HUVECs. (**a**) Volumetric view of the color-coded displacement map of the collagen gel structure by one vein endothelial growth factor (VEGF)-treated sprouting HUVEC. (**b**) Maximum projections of the displacement map (**a**) in each axis. (**c**) Normal and shear strain tensor of (**a**) as sliced along the XY plane, 45 microns from the top surface (median of the axial field of view). (**d**) Volumetric view of the color-coded displacement map of the collagen gel structure by two distant VEGF-treated sprouting HUVECs. (**e**) Maximum projections of the displacement map (**d**) in each axis. (**f**) Normal and shear strain tensor of (**d**) sliced along the XY plane, 45 microns from the top surface (all fields of view are 160 × 160 × 90 µm^3^).

The GD-OCM system was applied to identify the cellular physical interactions through the ECM medium in 3-D using a culture of HUVECs, which are the representative in vitro experimental model for studying vasculogenesis. First, one VEGF-stimulated HUVEC converged and caused displacement of the surrounding collagen gel structure toward the centrally located cell in all directions, as observed in 3-D (Figure 6a,b). This means that the cell was pulling the surrounding matrix inward, although the HUVEC branches were intruding outward into the collagen gel structure. The traction force of the cell produced a higher strain, especially near the branches, and each branch generated alternate contractile–extensible distortions of the surrounding ECM structure (Figure 6c).

Interestingly, two distant HUVECs showed magnetic field-like displacement of the collagen matrix between cells (Figure 6d,e). Mechanical communication, such as the source–sink relation between two cells via the ECM medium, alternately changed the direction of the displacement over time (Video S1). Like the circulating Yin-Yang symbol, the strain map alternately showed the contraction of the collagen gel structure around one cell and the relaxation of the structure around the other cell (Figure 6f). In summary, using the GD-OCM system with DVC analysis, we were able to visualize and analyze the cell–ECM physical interactions of the initial state of the sprouting EC and the more complex communication of distant cells through the ECM. Therefore, this GD-OCM system with DVC analysis can be a useful tool to characterize various biophysical mechanisms in experimental biology as well as cell and tissue engineering fields.

## 4. Discussion

ECM provides cells with a physicochemical environment for the mediation of essential cellular processes, including migration, proliferation, differentiation, and death [50,51,52]. Thus, understanding the physical interaction between cells and the ECM is crucial for elucidating the important biological mechanisms underlying development, morphogenesis, and tissue regeneration [53,54,55,56]. For this purpose, we introduced an imaging technique using GD-OCM by building on a previous study that used SHGM [44] as a technique for visualizing and analyzing the structure of collagen type I, a representative natural ECM component. The common advantage of these two optical technologies is that 3-D collagen gel structures with living cells can be visualized without the need for labeling or the use of fluorescently-tagged collagen proteins. Nevertheless, the reason for the introduction of the GD-OCM imaging system, in this study, was to allow the possibility of obtaining a 3-D full-field image at a much faster rate than the speed of changes occurring in cell, allowing them to be observed and recorded, in addition to the fact that this system is able to obtain 3-D images of a large area over long periods of time without inducing any cellular damage. In addition, when using low NA optical equipment for a broad field of view, SHGM has a disadvantage regarding the resolution of the axial axis, while GD-OCM can achieve isotropic resolution with little difference between the transverse and axial axes. Consequently, the introduction of the GD-OCM system has enabled us to improve on the shortcomings of SHGM.

Speckles, a feature of coherence imaging, are accompanied by noise and actual structural information of the media. Many techniques, such as image filtering, [57,58], artificial intelligence [59], or spectral compounding [60,61], have been reported to achieve visual improvements. Additionally, hardware-based methods such as optical chopper [62] or angular compounding [63] have been suggested for diminishing the effect of speckles. While the methods in this study reduced speckle noise, they also resulted in the loss of information on matrix deformation as well as had lower temporal resolution. Therefore, to quantify deformation of the collagen gel structure, we focused on finding optimal arbitrary volumetric patterns for efficient DVC analysis by comparing errors for various sampling rates. As a result, we determined that 600 sampling points (pixels) for 320 microns in the transverse field of view, which corresponds to 3.75 pixels per 2 μm (FHWM of transversal PSF), produces images within the optimal spacing range for DVC analysis (~3–5 pixels) [64]. While a lower error rate and higher accuracy of the DVC analysis could be achieved using a higher sampling rate, this would also decrease data throughput by increasing the amount of data and calculation time. Eventually, we were able to find an optimal sampling rate and quantify the collagen gel structure deformation in microscopic resolution.

From the results of the displacement of the collagen gel structure using DVC analysis, we calculated the strain tensor to interpret the deformation. The strain maps showed a heterogeneous response over each axis plane, as shown in Figure 5b. This heterogeneity may be caused by the structural characteristics of the porous fiber matrix or the local remodeling of the ECM. A more accurate and appropriate method of calculating strain and stress would be the application of the finite element method (FEM). However, when using natural polymers such as collagen gel, the physical properties vary as a result of various factors, such as concentration, pH, and polymerization temperature, so calibration should be executed for each sample. As a solution to this, one can consider the use of optical coherence elastography (OCE), a rheology technology based on OCT [65,66,67,68,69]. OCE can measure strain at a very high resolution through the measurement of phase variation with microscopic resolution, and physical properties can be estimated using information about the pre-external force. However, since the measurement of phase variation is still limited along the *Z*-axis, in the future, the use of an intensity-based method (DVC) to measure all 3-D variations would allow for the development of integrated micro-elastography with 3-D traction force microscopy using the OCT technique.

Another reason that the GD-OCM system is a great tool for observing biospecimens, including cells surrounded by the ECM, is that cells and the ECM can be simultaneously imaged without extra processing. To analyze the cell–ECM images taken with GD-OCM using DVC, it is necessary to first clearly segment the regions of the cell and ECM. Related methods, such as a k-means clustering-based approach [22], have been reported for the delineation of cell and ECM regions. In this study, the mean projection of the GD-OCM image for each axis, followed by intensity thresholding, was used to mask images for segmentation of cells and ECM regions.

Using HUVECs as an experimental model for vasculogenesis in this study, it is easy to observe the matrix remodeling that results from cells actively invading into the ECM according to the concentration of VEGF. To quantify the sprouting ability of HUVECs, many in vitro studies have simply counted the number of branches or measured the length of budding as the analytic methodology has been limited. Thus, we developed and applied an GD-OCM imaging system with DVC analysis to observe the interaction between a HUVEC and the ECM and confirmed that the cell was continuously pulling the ECM inwards, despite the increasing cell volume and the expansion of its branches outwards. This result is consistent with our previous research using SHGM, in which the physical interaction of mesenchymal stem cells with the surrounding ECM was quantified [44]. In particular, the visualization of two distant HUVECs communicating through the collagen gel structure via force was insightful. This deformation was a result of the force imbalance between cells when one cell pulls or releases the matrix more strongly than another cell. As such, the analysis of cell-induced matrix deformation suggests that novel findings, which are not possible with conventional biological methods, can be derived through physical interaction analysis, even in experiments with more complex conditions that mimic the in vivo environment.

## 5. Conclusions

An GD-OCM 3-D imaging system was developed to visualize the transparent natural ECM medium in microscopic resolution, which is difficult to observe with conventional microscopic equipment, and the DVC algorithm was efficiently applied following sampling rate optimization to quantify the deformation of the matrix structure by living cells. In addition, applying this approach to an experimental model of vasculogenesis, we could successfully visualize and analyze the cell–ECM physical interactions of the initial moment of HUVEC sprouting as well as the more complex communication between distant cells through the ECM. We believe that use of this GD-OCM imaging system with DVC analysis can be of great help in finding clues to reveal various biophysical mechanisms that have yet to be identified.

## Figures and Tables

**Table 1:** Error floor and displacement of measured deformation for various sampling rates.

Type	Error Floor STD ^1^ (IQR ^2^)			Displacement Mean (Median)			
Sampling Rate (µm/px)	0.8	0.53	0.4	0.8	0.53	0.4	GT^3^
U **(µm)**	0.111	0.047	0.103	3.195	3.199	3.199	3.20
	(0.147)	(0.071)	(0.160)	(3.198)	(3.200)	(3.199)	
V **(µm)**	0.068	0.040	0.208	3.195	3.199	3.199	3.20
	(0.094)	(0.060)	(0.377)	(3.198)	(3.199)	(3.199)	
W **(µm)**	0.150	0.162	0.201	4.417	4.423	4.417	4.42
	(0.220)	(0.209)	(0.313)	(4.422)	(4.424)	(4.419)	
Average **(µm)**	0.110	0.083	0.171	3.602	3.607	3.605	3.61
	(0.154)	(0.113)	(0.283)	(3.606)	(3.608)	(3.606)	

^1^ Standard deviation; ^2^ Interquartile range; ^3^ Ground truth.

## Supplementary Materials

The following are available online at https://www.mdpi.com/1996-1944/13/12/2693/s1, Figure S1: Transversal sampling rate optimization with a physically translated sample, Video S1: Dynamic interaction between two distant HUVECs through ECM over time, Source code S1: RANSAC plane fitting script for 3-D OCM image, and Source code S2: Visualization demo of DVC result with OCM image.
